# Rab21-Targeted Nano Drug Delivery System-Based FFPG for Efficient Paclitaxel Delivery to Inhibit Lung Cancer Progression

**DOI:** 10.3390/pharmaceutics17010094

**Published:** 2025-01-12

**Authors:** Jing Wang, Xueying Yan, Wenfei Wang, Shu Wang, Hongxiang Jiang, Xinhua Zhu, Zhehui Li, Defu Cai, Yonggang Xia

**Affiliations:** 1 Key Laboratory of Basic and Application Research of Beiyao (Heilongjiang University of Chinese Medicine), Ministry of Education, 24 Heping Road, Harbin 150040, China; wangjing@qmu.edu.cn (J.W.); yanxueying@hljucm.edu.cn (X.Y.); wwangsshu@163.com (S.W.); Jjianghongxiang@163.com (H.J.); 18904617802@163.com (X.Z.); zhehuili001@163.com (Z.L.); 2 Research Institute of Medicine and Pharmacy, Qiqihar Medical University, Qiqihar 161006, China; cai@qmu.edu.cn; 3 Bio-Pharmaceutical Lab, College of Life Sciences, Northeast Agricultural University, Harbin 150030, China; wangwenfei@neau.edu.cn

**Keywords:** fructooligosaccharides, fructans, *Platycodon grandiflorus*, lung cancer, liposomes, Rab21, Golgi-ER transport system

## Abstract

**Background/Objectives**: *Platycodon grandiflorus* (PG) has been widely researched as a conductant drug for the treatment of lung diseases by ancient and modern traditional Chinese medicine (TCM) practitioners. Inspired by the mechanism and our previous finding about fructans and fructooligosaccharides from *Platycodon grandiflorus* (FFPG), we developed a nano drug delivery system (NDDS) targeting lung cancer. The aim was to improve the efficiency of the liposomal delivery of Paclitaxel (PTX) and enhance the anti-tumor efficacy. **Methods**: The FFPG-Lip-PTX NDDS was prepared by electrostatic adsorption. Dynamic light scattering, zeta potential, and transmission electron microscopy were used for physical characterization. The release behavior of the NDDS was simulated by dialysis. The uptake of the NDDS was observed by confocal microscopy and flow cytometry. Cytotoxicity, apoptosis, migration, and invasion experiments were used to evaluate the anti-tumor ability of the NDDS in vitro. The penetration and inhibition of tumor proliferation were further analyzed via a 3D tumor sphere model. Finally, in vivo biological distribution and pharmacodynamic experiments verified the targeting and anti-tumor ability of the FFPG-Lip-PTX NDDS. **Results**: FFPG-Lip-PTX possessed a homogeneous particle size distribution, high encapsulation efficiency, and stability. In vitro experiments confirmed that FFPG promoted the uptake of the NNDS by tumor cells and enhanced cytotoxicity. It also increased the anti-tumor effect by promoting cell apoptosis and inhibiting invasion and metastasis. The same conclusion was obtained in 3D tumor spheres. In vivo experiments exhibited that FFPG-lips-PTX showed more significant lung cancer-targeting activity and anti-tumor effects. **Conclusions**: In this study, a novel lung-targeted NDDS is proposed to enhance the therapeutic effect of chemotherapy drugs on lung cancer.

## 1. Introduction

Lung cancer is the most common malignant tumor, and because of the high incidence, high metastasis rate, and high recurrence rate, it has been ranked as having the world’s highest cancer mortality rate [1]. Currently, the main therapeutic strategies encompass still surgical resection, chemotherapy, radiotherapy, and targeted therapy [2]. Among them, chemotherapy has an important impact on the prognosis and metastasis of lung cancer after comprehensive treatment [3]. Paclitaxel (PTX) is a broad-spectrum plant-based anticancer drug used to treat a variety of malignant tumors, including lung cancer. PTX is an antimicrocontroller that maintains tubulin stability during the treatment of tumors [4], while causing cell cycle arrest and inhibiting tumor cell division and proliferation through the combined action of the two mechanisms [5]. However, its water solubility is poor, which can affect the absorption and distribution of drugs, weaken anti-tumor activity, and can produce histamine in the degradation in vivo, increasing allergic reactions and peripheral neurotoxicity [6,7]. These side effects suggest that the benefits gained from PTX appear to have reached a plateau.

Multidisciplinary integration provides a new strategy to address the above problems. With the development of nanotechnology, research about nano drug delivery systems (NDDSs) for the delivery of anticancer drugs has achieved excellent results [8]. Among the research findings, liposomes have been successfully applied in clinics due to their special effect of fusing with the cell membrane and improving the solubility [9] and bioavailability of drugs [10]. Therefore, the selection of liposomes as the drug carrier not only reduces the toxicity of PTX, but also improves the anti-tumor efficacy by enabling the drug to be fully absorbed due to its high cell affinity [11,12]. However, liposomes are easily captured and removed by the reticuloendothelial system, preventing the drug from reaching the lesion site. Therefore, the specific recognition of overexpressed receptors or epitopes on target cells by liposome targeting is a key strategy to address the above challenges.

Oligosaccharides from traditional Chinese medicine (TCM) can be taken up by tumor cells through specific mechanisms and exert their special biological functions [13]. A compound anticancer Chinese medicine oral liquid forms positively charged alkaline nanoshells by grafting drug molecules onto chitosan oligosaccharides. These nanoshells can be absorbed across the membrane into the bloodstream and bind to negatively charged tumor cells, being taken up by the tumor cells into the cell interior [14,15]. This indicates that TCM oligosaccharides can be taken up by tumor cells and support the potential application of TCM oligosaccharides in anti-tumor treatments.

Platycodonis Radix (the root of *Platycodon grandiflorus*) (PG) is a well-known traditional Chinese medicine (TCM), and as an important lung conduction drug, is widely used in the treatment of lung diseases [16,17]. To date, several research projects have reported that PG maintains high intracellular drug concentrations due to the surface-active effect and inhibition of pump protein expression [18]. Our previous work started from the theoretical basis of traditional Chinese medicine (TCM), elaborated the modern pharmacological mechanism and material basis of PG as a guide drug, and successfully discovered a kind of fructooligosaccharide and fructogen of PG (FFPG) with a natural lung targeting ability [19]. It was also confirmed that FFPG promoted the movement of biomolecules to the target lung [19,20]. More importantly, we found that Rab21 was the core affinity target of FFPG [20]. Rab21 belongs to the GTPase monomer of the Rab family and is involved in the control of cell membrane transport and endocytosis [21]. The complex regulatory network formed by Rab GTPase to regulate vesicle transport in animal cells is centered on Rab21 [22]. It has been confirmed that the membrane protein Rab21 has a significantly high expression in the lungs [23]. As an undiscovered core affinity target, FFPG positively up-regulated endocytosis and increased extracellular substance uptake via the specific binding and activation of Rab21 on lung cells and tissue surfaces.

**Scheme 1:**
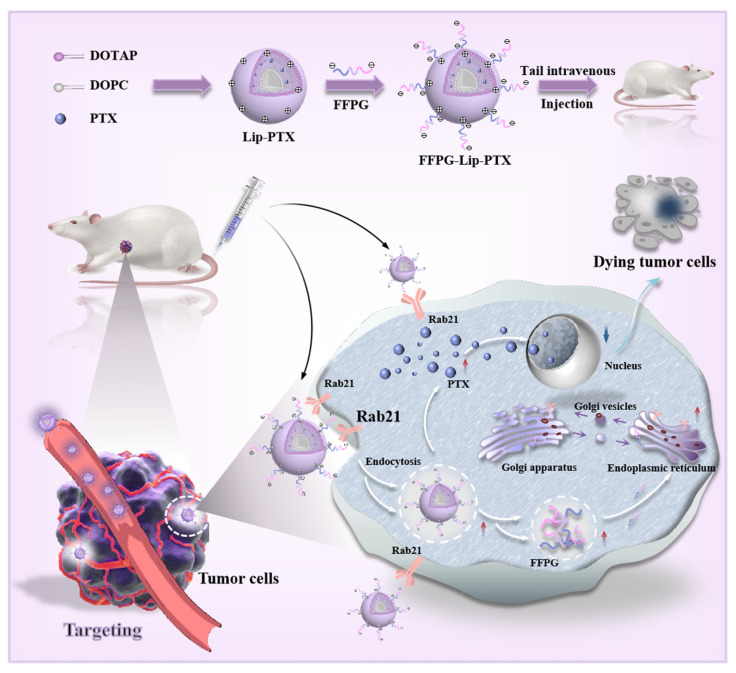
Scheme of FFPG-modified liposomes for targeted delivery of PTX in vivo.

The more significant finding was that there was a significant correlation between Rab21 and extracellular substance uptake by activating the Golgi-ER transport system. The results revealed that the Golgi and ER were successively activated by FFPG. The efficiency of extracellular substance uptake by cells was related directly and closely to the degree of Golgi-ER activation by FFPG [20]. Overall, our previous work mapped a Rab21-mediated vesicle transport pathway to explain the origin of the lung-directed guidance and targeting of FFPG based on the Golgi-ER transport system. It provided a material basis for the accurate development of new lung-targeted therapy strategies in the later stage. Considering these unique advantages of FFPG, we hypothesized that FFPG-modified liposomes may lead to improved circulatory stability and the preferential accumulation of tumor tissue, providing a desirable curative effect for improving the existing clinical therapeutics (Scheme 1).

## 2. Materials and Methods

### 2.1. Materials

1,2-Dioleoyl-3-trimethylammo-nium-propane (chloride salt) (DOTAP), Choline, 1, 2-dioleyl-SN-glycerol-3-phosphate (DOPC) were purchased from AVT Co., Ltd. (Shanghai, China). Egg yolk phosphatidylcholine (EPC), LIPOID E-80 was provided by LIPOID GmbH (Ludwigshafen, Germany). Paclitaxel (PTX > 99%) was purchased from Meilun Biotechnology Co., Ltd. (Dalian, China). FFPG was provided by Professor Yonggang Xia’s Laboratory of Chinese Medicine Chemistry, Heilongjiang University of Chinese Medicine. Agarose, Coumarin-6 (Cou6), Hoechst 33258, Sulforhodamine B (SRB), 1,1’-dioctadecyl-3,3,3’,3’-tetramethylindotricarbocyanine iodide (DiR), and matrigel were purchased from Sigma-Aldrich (Shanghai, China).

### 2.2. Cell Culture and Animals

Human NSCLC A549 cells were maintained in DMEM/F12 medium (Meilun, China) and mouse Lewis lung carcinoma cells (LLCs) were cultured in RPMI-1640 medium, which was obtained from Gibco (Shanghai, China). The culture medium was supplemented with 10% fetal bovine serum (FBS), which was purchased from Wisent Co., Ltd. (Nanjing, China), 100 U/mL penicillin and 100 µg/mL streptomycin, which were obtained from Macgene (Beijing, China). Cells were cultivated in a humidified incubator at 37 °C in 5% CO_2_. C57BL/6 mice, weighing 16–20 g, were supplied by Vitonglihua Co., Ltd. (Beijing, China).

### 2.3. Preparation of Drug-Loaded Liposomes

The drug-loaded liposomes were prepared as described previously, through thin-film evaporation and the ultrasonic technique. Briefly, DOTAP, DOPC, cholesterol, and EPC were dissolved in dichloromethane at a molar ratio of 1:1:1:2, and PTX was dissolved in methanol. Then, the solvent was mixed and evaporated by a rotary evaporator at 37 °C for 1 h to form a solid film. PTX was automatically loaded into the hydrophobic core of the Lip due to its hydrophobic nature in this process. The resulting thin film was hydrated in distilled water at 60 °C for 10 min to obtain PTX-coated liposomes. To prepare FFPG-coated liposomes (FFPG-Lip-PTX), gradient concentrations of FFPG solutions were quickly added into liposomes (1:1, *v*:*v*) under stirring at 37 °C. Stirring was maintained for another 10 min after the completion of the addition.

Similarly, liposomes loaded with Cou6 or DiR, FFPG-Lip-Cou6 and FFPG-Lip-DiR, were fabricated by the same method, where the PTX was replaced with Cou6 or DiR. The un-loaded PTX or fluorescent probes were separated from liposomes using a Sephadex G-50 gel column.

### 2.4. Characterization of Drug-Loaded Liposomes

The particle sizes, polydispersity indexes (PDIs), and zeta potential values of all NDDSs were determined by the Nicomp 380 ZLS particle sizing system (PSS, USA). The structures and morphology of liposomes were evaluated using transmission electron microscopy (TEM). Each sample (0.5 mg/mL) was negatively stained using a 2% phosphotungstic acid solution and characterized over a carbon-coated copper TEM grid (JEOL JEM-1200EX, Tokyo, Japan)

The lipids loaded with PTX were suspended in an acetonitrile solution and treated with sonification for 20 min using the FS60 bath sounder. Centrifugation at 3000 rpm for 10 min removed the undischarged PTX, and HPLC was used to quantify the free PTX (Shimadzu, Japan). The drug encapsulation efficiency was calculated according to the following encapsulation efficiency equation: (%) = (total PTX -unloaded PTX)/total PTX × 100.

Lip-PTX and FFPG-Lip-PTX were placed in PBS containing 50% FBS, and the distribution was observed at room temperature for 72 h to evaluate the serum stability.

### 2.5. In Vitro Drug Release Evaluation

The drug release profiles from the NDDSs were evaluated using a dialysis method. A total of 1mL Lip-PTX and FFPG-Lip-PTX were separately transferred into dialysis bags (MWCO 8000–14000) with a 30 mL release medium containing 0.5% Tween 80 and 0.5 M sodium salicylate. The samples were shaken at 100 rpm at 37 °C. At predetermined time points, 1 mL of the release medium outside the bag was collected and an equal volume of fresh media was supplemented. PTX concentrations were determined by HPLC (Shimadzu, Japan). Using an RP LiChrospher^®^ C18 column (100, 250 mm × 4.6 mm, 5 μm; Merck, Darmstadt, Germany), the column temperature was maintained at 35 °C. The mobile phase was acetonitrile to water in a ratio of 65:35. The detection wavelength was 227 nm. According to the standard curve of PTX, the cumulative drug release can be calculated.

### 2.6. In Vitro Cellular Uptake and Targeting Effects

To observe the cellular uptake and targeting effect of FFPG in A549 and LLC cells, Cou6 as a fluorescent probe was encapsulated in the liposomes. The density of 5 × 10^4^ cells per well were cultured in a 6-well plate overnight at 37 °C. Then serum-free medium containing the same concentration of free Cou6, Lip-Cou6, or FFPG-Lip-Cou6 was added and incubated at 37 °C for 2 h, then rinsed with cold PBS 3 times to terminate the uptake behavior of the cells. The mean fluorescence intensity of Cou6 was analyzed using a FACScan flow cytometer (BD FACSCalibur, Franklin Lakes, NJ, USA) with 1 × 10^4^ cells collected, excitation at 488 nm, and detection at 560 nm.

Confocal microscopy was used to visualize cell uptake. The experimental procedure was the same as above. After washing with cold PBS, cells were fixed with 4% paraformaldehyde for 15 min. The nuclei were stained with Hoechst 33258 for 30 min. Finally, The intracellular localization of nanoparticles was observed and analyzed using the LSM710 laser confocal microscope (Zeiss, Jena, Germany).

### 2.7. In Vitro Inhibition of Cell Proliferation

A549 cells and LLC cells were seeded into 96-well plates at a density of 3 × 10^3^ cells/well and preincubated overnight at 37 °C with 5% CO_2_. The Lip-PTX, FFPG-Lip-PTX, and free PTX solutions at serial PTX concentrations (2.5–320 ng/mL) were added for 48 h (n = 6) separately. The medium in each wall was removed and the cells were fixed with 10% cold trichloroacetic acid, then stained with 0.4% SRB to quantify the living cells. Finally, the absorbance of the final solution representing cell viability was detected by a multifunctional microplate reader (Tecan Safire2, Switzerland) at 540 nm.

### 2.8. In Vitro Apoptosis-Inducing Effects

Apoptotic cell death was analyzed by Annexin V-FITC/7AAD staining using a flow cytometer. Briefly, A549 cells or LLC cells were seeded in a 6-well culture plate at a density of 5 × 10^5^ cells/well. After incubation overnight, the cells were treated with fresh medium PTX liposomes (as a blank control) for 24 h. The concentration of all PTX-loaded formulations contained the equivalent PTX of 30.0 ng/mL. Then, the cells were harvested and suspended in the provided binding buffer and stained with Annexin V-FITC and PI at room temperature in the dark for 15 min away from light. The cells were immediately analyzed using a FACScan flow cytometer with the events collected 1 × 10^4^. Each assay was carried out in triplicate.

### 2.9. In Vitro Cell Migration and Invasion Assay

To quantitate vertical motility, an in vitro transwell migration assay was used. For the migration assay, the A549 cells were incubated in the PTX-loaded liposome culture system with the same concentration for 24 h. After digestion, 5 × 10^4^ cells in 100 μL serum-free medium were seeded in the upper chamber of the transwell (24-well insert; 8 μm pore), with 600 μL of complete media added to the lower chamber. After 24 h of incubation, non-migration cells were removed. Cells that migrated through the membrane were washed with PBS and fixed with 4% paraformaldehyde (*v*/*v*) at room temperature, stained with crystal violet (1 mg/mL) for 20 min, and counted by microscopic examination. Finally, crystal violet was dissolved and the absorbance at 570 nm was measured by an enzyme-labeled instrument.

Similarly, cell invasion activity was measured by a transwell Matrigel invasion assay as described previously [24]. Each well was pre-coated with 50 micomoles of Matrigel (BD Bioscience) (remove stop) before the invasion assay. The subsequent evaluation procedures corresponded to the above-described migration method.

### 2.10. Effect on Uptake and Proliferation of Three-Dimensional Tumor Spheroids

The suspension drop system was adopted to culture the 3D multicellular tumor spheroids of A549 cells [25]. Briefly, at first, the bottom of the 48-cell plate was coated by a sterilized agarose solution (2% *w*/*v*) to prevent cellular attachment. Then, 1000 cells in the 20 μL complete medium was suspended on the lid of well. After 3 days of cell growth, the formed tumor spheroids were transferred into the plate, and the medium containing the same concentration of PTX or Cou6 delivery system was added to each pore. The penetration effect of tumor spheroids was evaluated by LSCM.

The growth of tumor spheres was observed under an inverted microscope daily, and the proliferation inhibition of tumor spheres was evaluated by calculation. The volume was calculated by using the following formula:

V = 1/2 × *d*_length_ × *d*_width_^2^

The selected tumor spheres were subsequently used for penetration experiments. Briefly, spheres were incubated with Cou6 liposomes (Cou6 concentration was 100 ng/mL) for 6 h, respectively. Then, cell spheres were washed with ice cold PBS gently before being transferred to confocal dishes and analyzed using a confocal laser scanning microscope. Z-stack images were obtained by scanning the spheres at the different layers to investigate the penetration ability of liposomes.

### 2.11. In Vivo Imaging and Biodistribution Analysis

In order to investigate the ability of FFPG-modified liposomes to target lung cancer grafts, a lung cancer bearing C57BL/6 mice model was established via subcutaneous injection of LLC-Luc cells (5 × 10^5^) into the right flanks. Mice with a tumor volume around 400 ± 100 mm^3^ were divided randomly into 2 groups (n = 3) and injected intravenously with Lip-DiR or FFPG-Lip-DiR (100 μL, DiR: 1 mg/kg) via the tail vein. After injection, the live imaging of small animals was performed at different time points, and mice were dissected 48 h later to harvest major organs for ex vivo imaging to investigate the distribution of liposomes. During the experimental process, isoflurane inhalation was used to anesthetize the mice. At the end of the experiment, cervical dislocation was used to euthanize the mice.

### 2.12. In Vivo Anti-Tumor Efficacy and Targeting Mechanism

A xenograft tumor model was established as described above to evaluate the tumor inhibitory rate (TIR). When the volumes of tumor tissues reached about 100 mm^3^, the mice were selected and randomly divided into 4 groups (n = 7). Then, the mice were injected with physiological saline (Control), free PTX, Lip-PTX, or FFPG-Lip-PTX solution via the tail vein every two days (10 mg/kg for PTX). During the 10-day treatment cycle, the tumor volumes and changes in body weight of each mouse were monitored and recorded regularly. At the end of the treatment period, mice were deeply anesthetized, and blood was collected from the eyeballs, with approximately 1 mL of blood taken. After the mice died, the heterotopic transplanted tumors were excised and weighed, and the main organs were collected. All the above operations were carried out under the approval of the Experimental Animal Ethics Committee of Heilongjiang University of Chinese Medicine. Then, all excised tissues were fixed in 4% paraformaldehyde for systematic pathological analysis, including H&E, IHC staining, and TUNEL. Finally, the positive expressions of Rab21, endoplasmic, and Golgi in the four groups of tumor tissues were detected by IHC.

### 2.13. Statistical Analysis

All experiments were repeated at least three times. Statistical significance between groups was determined using the student’s t-test. The data are presented as mean ± SD, and *p* ≤ 0.05 was considered significant.

## 3. Results and Discussion

### 3.1. Preparation and Characterization of FFPG-Lip-PTX

**Figure 1:**
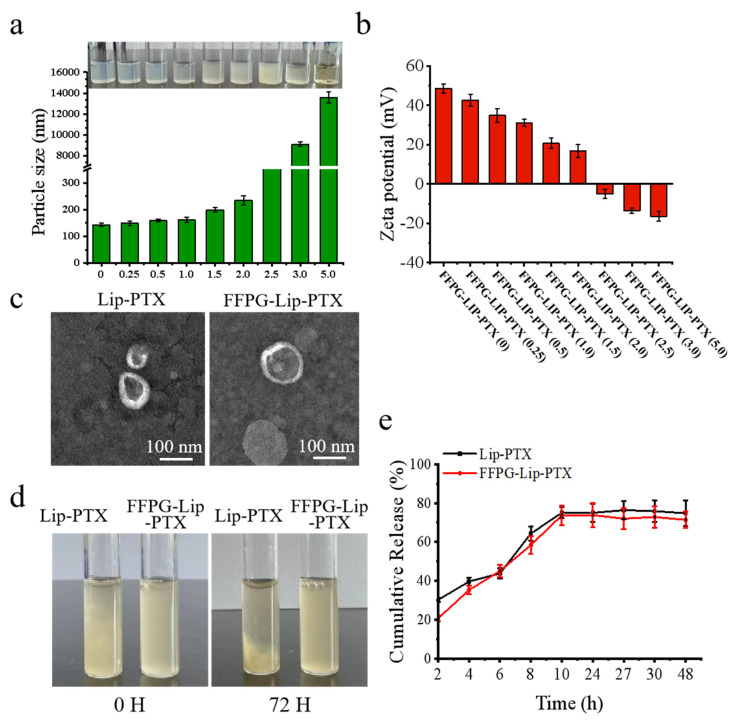
Preparation and characterization of the physical and chemical properties of FFPG-Lip-PTX. (**a**) The representative images of FFPG-Lip-PTX at different FFPG concentrations and effect of FFPG concentration on FFPG-Lip-PTX particle diameter. (**b**) Effect of FFPG concentration on FFPG-Lip-PTX zeta potential. (**c**) TEM image of Lip-PTX and FFPG-Lip-PTX. (**d**) Stability studies of FFPG-Lip-PTX and Lip-PTX after incubation with PBS containing 50% FBS at 37 ℃ for 72 h. (**e**) The in vitro cumulative release profile of PTX from the Lip-PTX and FFPG-Lip-PTX at 37 ℃ during 48 h. Data are shown as mean ± SD (n = 3).

Cationic liposomes which encapsulated PTX were prepared using the hydration method. The surface of the liposomes was modified by linear polysaccharide FFPG with a negative charge to prepare the FFPG-Lip-PTX NNDS, which avoided the toxicity of covalent modification by chemical reaction [26]. The optimum reaction conditions for FFPG-Lip-PTX were determined by particle size distribution. When the FFPG concentration was lower than 1.0 mg/mL, the particle size was stable at about 150 nm, and gradually increased with the continuous increase in the concentration (Figure 1a). When the FFPG concentration was greater than 3.0 mg/mL, the particles aggregated and sank, during which the zeta potential values changed sharply from +48.50 mV to +21.19 mV, and the stable system of electrostatic repulsion between liposomes could not be maintained (Figure 1b). Changes in the charge of liposomes indicated that FFPG was successfully adsorbed on Lip-PTX [24,27]. To balance long-period nanoparticles of an ideal size with sufficient ligands to target lung cancer, the concentration of FFPG was 1.0 mg/mL. The PDI was 0.218 ± 0.040 and the particle size of FFPG-Lip-PTX was 184.2 ± 2.1nm. The optimal particle size is in the range of 20−200 nm, making the liposomes strong enough to withstand fluid flow (such as blood and lymph), while allowing the liposome particle to pass through the interstitium [28]. The zeta potential was +31.13 ± 0.15 mV, the positive charge avoided electrostatic repulsion with the cell membrane [29], and the absolute potential value of liposomes was greater than 30 mV, forming a stable system and avoiding the accumulation and deposition of liposomes on the vascular wall [30]. TEM images showed that Lip-PTX and FFPG-Lip-PTX were uniformly distributed spherical particles with a double-layer membrane structure (Figure 1c). This was consistent with particle size measurements. FFPG-Lip-PTX remained stable at room temperature for 72 h and, relatively speaking, Lip-PTX accumulated precipitation (Figure 1d). The EE of the optimized liposomes against PTX ranged from 80.8% to 91.5%. In summary, the transformation of the particle size and surface charge confirmed that the anionic lung-targeting polysaccharide FFPG was successfully loaded on the surface of liposomes and formed a stable system.

### 3.2. Stability and In Vitro Drug Release of FFPG-Lip-PTX

The PTX release of Lip-PTX or FFPG-Lip-PTX was measured by dialysis, and the release curve is shown in Figure 1e. As shown in the curve, under the simulated humoral environment of 0.5 M sodium salicylate and PH 7.4, both Lip-PTX and FFPG-Lip-PTX liposomes showed a slow and sustained release process. The cumulative release of PTX basically reached the maximum release rate within 10 h, at which time their release rates were 75.08% and 73.03%, respectively. There was no significant difference between them. Due to the decrease in lipid bilayer fluidity and membrane permeability, no significant extracellular PTX leakage occurred in vivo. The results showed that FFPG modification did not affect the drug release advantage of liposomes, which provided a guarantee for the delivery of PTX by FFPG-modified liposomes for anti-lung cancer research in vivo and in vitro.

### 3.3. Cellular Uptake and Targeting Effects

**Figure 2:**
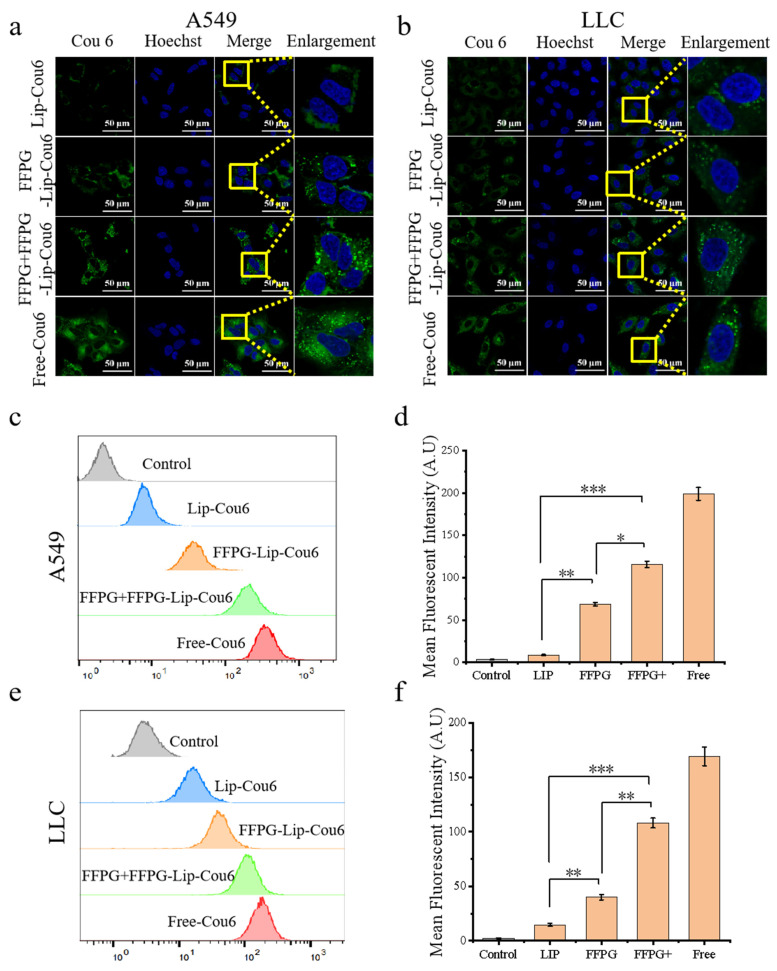
Evaluation of extracapsular efficacy of FFPG-Lip-PTX liposomes on A549 and LLC cells after incubation for 1 h at 37 ℃. The final concentration of Cou6 was 100 ng/mL. Laser scanning confocal microscopy images of A549 (**a**) and LLC cells (**b**) incubated with different formulations. Green and blue indicate the fluorescence of Cou6 and Hoechst 33258, respectively. Flow cytometric quantitative determination of Cou 6 uptake on A549 cells (**c**) and LLC cells (**d**). Quantitative analysis of Cou6 uptake based on flow cytometric plots on A549 (**e**) and LLC cells (**f**). Each bar represents the mean fluorescence intensity ± SD (n = 3), * *p* < 0.05, ** *p* < 0.01, ***p < 0.001.

Enhanced PTX delivery via surface-modified FFPG via liposomes was confirmed in A549 and LLC cancer cells overexpressing Rab21 [23,31]. To visualize the cellular uptake and intracellular distribution of liposomes under CLSM, the fluorescent dye Cou6, which replaced PTX, was loaded into the liposomes. As shown in Figure 2a,b, cells treated with FFPG-LiP-Cou6 showed significantly higher green fluorescence than Lip-Cou6, indicating the effect of Rab21 on the lung targeting of FFPG, and FFPG-LiP-Cou6 liposomes were internalized into the Golgi-ER of cancer cells. In contrast, cells incubated with Lip-Cou6 showed very weak Cou6 fluorescence, due to a much lower uptake of Lip-Cou6 by cells. It is worth noting that the FFPG pre-incubated group exhibited the strongest fluorescence intensity except for free Cou6. This reconfirmed our previous findings that FFPG targets lung cancer through its affinity for Rab21 and activates the Golgi-ER transport system to enhance the NDDS.

In addition, quantitative flow cytometry confirmed that nearly all A549 and LLC cells became Cou6 fluorescence-positive after FFPG-Lip-Cou6 incubation, while the fluorescence intensity of Cou6 was relatively weak (Figure 2c–f). It is well-known that positively charged surfaces can promote the endocytosis of the NDDS through electrostatic interactions with negatively charged cell membranes [32]. More importantly, FFPG activated the Golgi-ER and promoted the co-localization of PTX with the Golgi-ER in lung cancer cells, thus significantly increasing the uptake and distribution of PTX in lung cells and tissues (here, Cou6 serves as an analogue). In contrast, the uptake efficiency of Lip-Cou6 was relatively low. Previous research had found that the activation process of FFPG on the Golgi-ER was highly time-related, reaching a peak at 9 h, which was well demonstrated by the pre-incubation experiments of FFPG with A549 or LLC lung cancer cells designed by us (the relative fluorescence intensity of FFPG + FFPG-Lip-Cou6 group was significantly higher than that of the other two Lip groups). These results further rationalized the structural design of our FFPG liposomes, with the first stage of extracellular FFPG targeting acting on the stable delivery of liposomes to lung cancer cell sites, and the second stage of PTX being released within cancer cells to play a chemotherapeutic role. This advantage ensures that our NDDS achieves a more effective anti-tumor impact.

### 3.4. In Vitro Cytotoxicity and Apoptosis Assay

**Figure 3:**
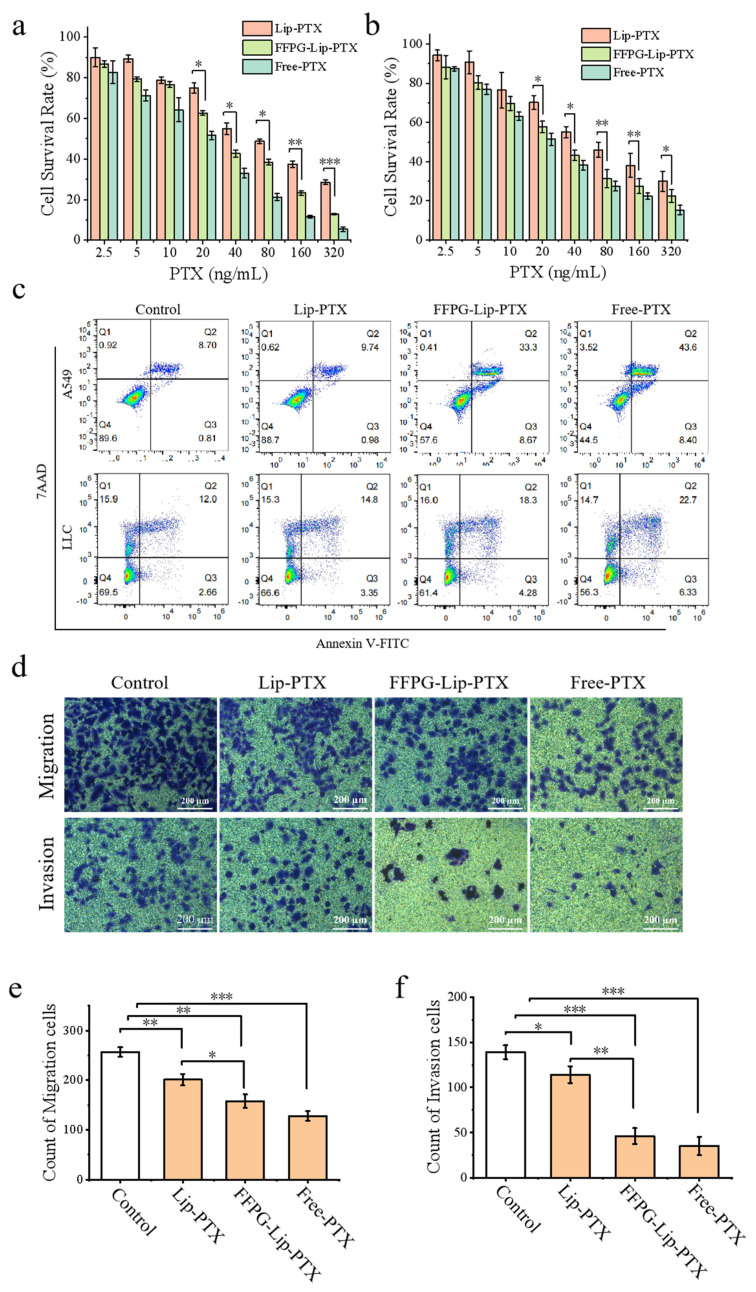
Evaluation of extracapsular efficacy of FFPG-Lip-PTX liposomes. The in vitro cytotoxicity of different PTX preparations to (**a**) A549 and (**b**) LLC cells was determined by the SRB method. (**c**) In vitro apoptosis of different PTX preparations in A549 and LLC cells was evaluated by flow cytometry. (**d**) In vitro inhibition of A549 cell migration and invasion by different PTX preparations observed using transwell images (30 ng/mL). Quantitative inhibition of A549 cell migration (**e**) and invasion (**f**). Data are expressed as mean ± SD (n = 3), * *p* < 0.05, ** *p* < 0.01, *** *p* < 0.001.

The cytotoxic effect of FFPG-Lip-PTX on tumor cells was significantly superior to that of Lip-PTX. This is because PTX was more efficiently delivered to lung cancer cells by directly binding to Rab21 to accelerate the uptake of extracellular therapeutic substances (Figure 3a,b). IC50 values of Lip-PTX and free PTX were 53.2 ng/mL and 28.5 ng/mL in A549 cells, and 50.6 ng/mL and 27.3 ng/mL in LLC cells, respectively. The IC50 value of FFPG-Lip-PTX was further reduced to 35.5 ng/mL (A549) or 36.1 ng/mL (LLC). This implies that merely a low dose of FFPG-Lip-PTX can achieve the same anti-tumor effect as a high dose of Lip-PTX. The results suggested that FFPG-mediated active targeted delivery reduced the dose of PTX required to achieve anticancer activity on lung cancer cells. The aim of reducing toxicity and increasing the efficiency of TCM theory was realized.

The flow cytometric analysis of cell apoptosis provided consistent results (Figure 3c).The A549 and LLC cells incubated with various liposomes at the same PTX concentration of 30 ng/mL displayed different apoptosis levels. The pulmonary targeting of FFPG was essential for PTX-loaded liposomes to induce apoptosis. Due to poor cell uptake, Lip-PTX only induced apoptosis in 9.74 ± 0.05% and 14.80 ± 0.08% of cells. By comparison, FFPG-Lip-PTX showed a stronger effect in inducing apoptosis (33.30 ± 0.09% and 18.30 ± 0.06%), which was due to the efficient delivery of PTX to A549 and LLC cells via FFPG-modified lung targeting liposomes. Free PTX showed the strongest apoptosis-inducing level in vitro (43.60 ± 0.11% and 22.70 ± 0.08%), which was because of the effect of free diffusion.

### 3.5. Valuation of Cell Migration and Invasion

Furthermore, we explored the ability of FFPG-Lip-PTX in suppressing cancer cell migration and invasion. Migratory and invasive abilities are critical in the metastatic cascade [33]. Tumor cells with FFPG-Lip-PTX indicated remarkably decreased cell migration when compared with control and Lip-PTX group via the transwell assay (Figure 3d,e). In Lip-PTX group, the migration ratio was 63.8 ± 0.5% after 24 h, but this was significantly reduced to 30.3 ± 0.7% by the treatment with FFPG-Lip-PTX, and further reduced to 22.8 ± 0.1% with free PTX. Next, Matrigel was used to simulate the extracellular matrix of the tumor tissue for the transwell invasion experiment [34]. A similar trend as the migration assay is shown in Figure 3d,f; FFPG-Lip-PTX reduced the invasion over the Matrigel-coated membrane by 45.2 ± 0.9%, Lip-PTX by 63.5 ± 0.4%, and free PTX by 38.4 ± 0.6%. Following modification with FFPG, the inhibitory effect on tumor metastasis in vitro was significantly improved, which may be related to FFPG-Lip-PTX generating more PTX uptake, making it a potential excellent suppressor of tumor metastasis.

### 3.6. Penetration and Growth Inhibition on 3D Tumor Spheroid

**Figure 4:**
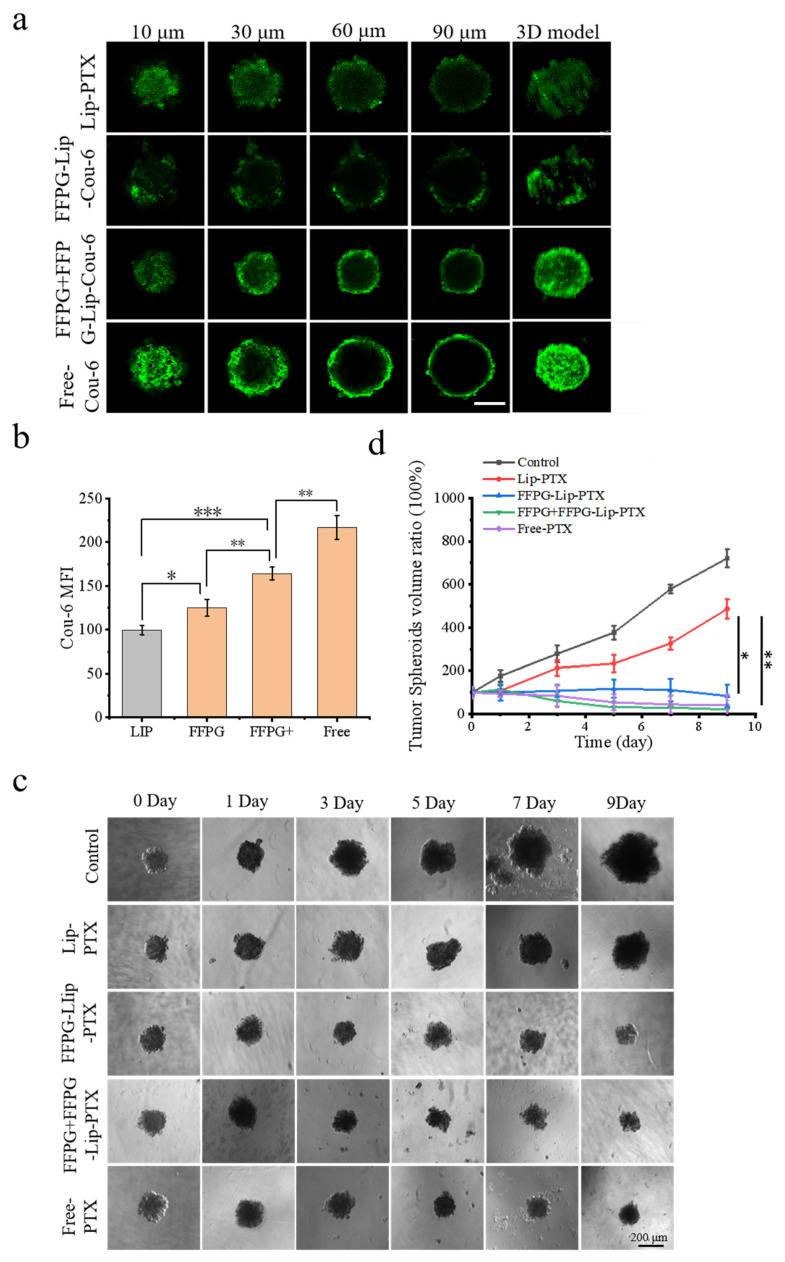
Effects of different Cou6 and PTX preparations (PTX = 40 ng/mL) on the penetration and growth of A549 tumor spheres. (**a**) Silver light image of penetration intensity of different Cou6 preparations on 3D tumor spheres (sale bar, 50 μm). (**b**) Semi-quantitative penetration strength of different Cou6 preparations on tumor spheres. (**c**) Image of a typical tumor sphere under an inverted microscope. (**d**) Quantified size of tumor spheres at different time points. Data are expressed as mean ± SD (n = 3), * *p* < *0*.05,** *p* < 0.01, *** *p* < 0.001.

In this study, A549 cell spheres were selected as the experimental model, the green fluorescence probe Cou6 was used as the marker, and the fluorescence intensity of tumor-like multicellular spheres at different depths was observed by laser confocal microscopy to simulate the penetration ability of different preparations in solid tumors. As shown in Figure 4a,b, the fluorescence intensity of each group was free Cou6, FFPG+ FFPG-Lip-Cou6, FFPG-Lip-Cou6, and Lip-Cou6, in order from high to low. This can be explained by the fact that free Cou6 enters cells in a free diffusion way and is relatively easily taken up by cells [35]. While the NDDS FFPG-Lip-Cou6 was taken up by cells through the specific targeting effect of Rab21 and the cytoendocytosis of liposomes, its uptake efficiency was higher than that of passive Lip-Cou6. It is worth noting that the pre-incubation of the FFPG + FFPG-LiP-Cou6 group stimulated the key activation of the Golgi-ER transport effector, accelerated the drug delivery system’s entry into lung cancer cells, and thus obtained the largest penetration ability of lung cancer solid tumor balls except for the free group, which further confirmed the previous experimental results. It also further confirmed the pulmonary targeting of FFPG due to its affinity for directly targeting Rab21.

The in vitro 3D tumor spheroid models contain the organized extracellular matrix with oxygen, nutrients, and energy gradients similar to those in vivo, thus exhibiting characteristics similar to solid tumors in vivo [36]. To further validate the effect of FFPG modification on anti-tumor activity, we used A549 tumor spheres to evaluate the 9-day growth inhibition. Figure 4c,d shows representative optical images of A549 tumor spheres treated by various PTX solutions at different time points. On the 9th day of tumor ball growth, the tumor ball volume ratio in the control group was 32.2 ± 26.8%, and Lip-PTX could reduce the volume by 51.3 ± 3.9%, which was significantly lower than that in the control group. FFPG-Lip-PTX had a better effect on the reduction in tumor sphere volume, which was 21.9 ± 1.1% after 9 days of treatment, and free PTX showed a stronger inhibitory effect on the tumor sphere due to the free diffusion effect. These results indicate that different PTX formulations could inhibit the growth of tumor spheres. It is worth noting that FFPG + FFPG-Lip-PTX showed the highest efficiency in inhibiting tumor proliferation. This may be related to FFPG modification improving the penetration and accumulation of liposomes into tumor spheres. It can be seen that FFPG pre-incubation activates Golgi-ER transport and enhances the uptake efficiency of drug-carrying liposomes by tumor spheres, indicating superior anti-tumor benefits brought by FFPG modification.

### 3.7. In Vivo Investigations of Biodistribution Utilizing Fluorescence Imaging Techniques

**Figure 5:**
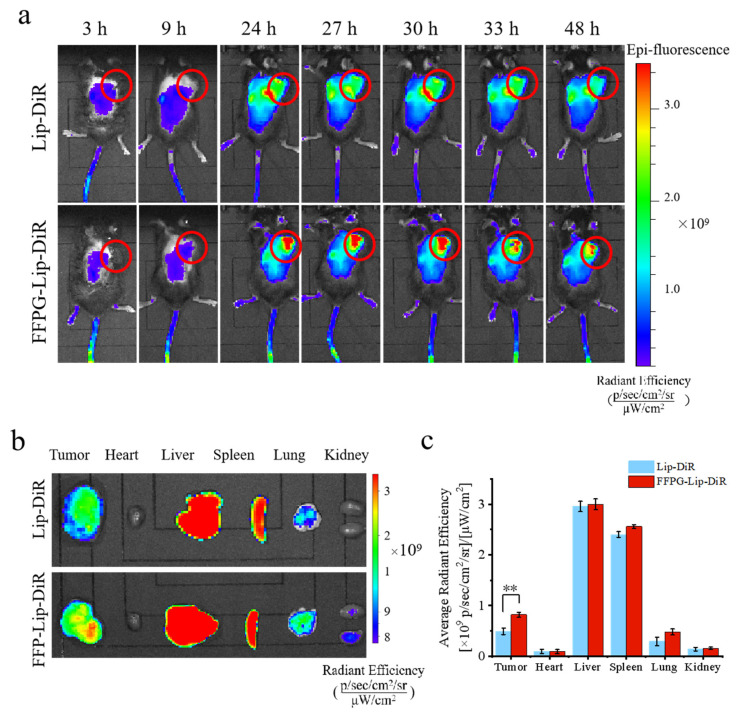
Biological distribution of Lip-DiR and FFPG-Lip-DiR in LLC tumor-bearing mice. (**a**) LLC real-time imaging of the tail vein of tumor-bearing mice within 48 h. (**b**) Fluorescence images of tumors and organs in vitro. (**c**) Semi-quantitative analysis of fluorescence intensity at tumor site. Data are expressed as mean ± SD (n = 3), ** *p* < 0.01. Tumors are represented by red ovals.

To further investigate the lung-targeting capability of FFPG-Lip in vivo, near-infrared fluorescent dye DiR was loaded into liposomes instead of PTX to monitor the distribution and tumor accumulation of liposomes in LLC xenograft C57BL/6 mice based on Small Animal In Vivo Imaging (SAIVI) technology. As shown in Figure 5a, 3 h post-injection was designated as the initial observation point. At this time, the accumulation of liposomes at the tumor site was not obvious in both groups. At 9 h after injection, the advantage of the accumulation in the tumor site of the FFPG-Lip-DiR group was noticeable. At 24 h post-injection, the accumulation advantage of FFPG-Lip-DiR at the tumor site became significantly pronounced and persisted until 48 h, when the tumor fluorescence attenuation rate of mice receiving FFPG-Lip-DiR was still slower than that of mice receiving Lip-DiR. This series of observations showed that FFPG modification greatly improved the accumulation efficiency and residence time of liposomes in tumors. It is suggested that FFPG specifically targets lung cancer tissue. Our previous research on the properties and mechanisms of FFPG as a pulmonary “meridian” drug are further confirmed here.

The ex vivo fluorescence imaging results of the major organs and tumors of mice sacrificed at 48 h post-injection were consistent with the above results (Figure 5b). The fluorescence intensity of tumors in the FFPG-Lip-DiR group was 2.0 times that of the Lip-DiR group (Figure 5c), which further demonstrates the better tumor-targeting effect of FFPG-Lip-DiR. Attention was paid to the lung tissue. After 48 h of metabolism, less fluorescein remained in the lung, but the intuitive phenomenon indicated that the fluorescence intensity of the lung tissue in the active FFPG-Lip-DiR group was still higher than that in the passive Lip-DiR group. Using the SAIVI results in combination, we propose that FFPG obviously targets the lung, but not other organ tissues. Importantly, this unique specificity provides the possibility for the further development of targeted therapeutic drugs for lung cancer.

### 3.8. In Vivo Anti-Tumor Efficacy

**Figure 6:**
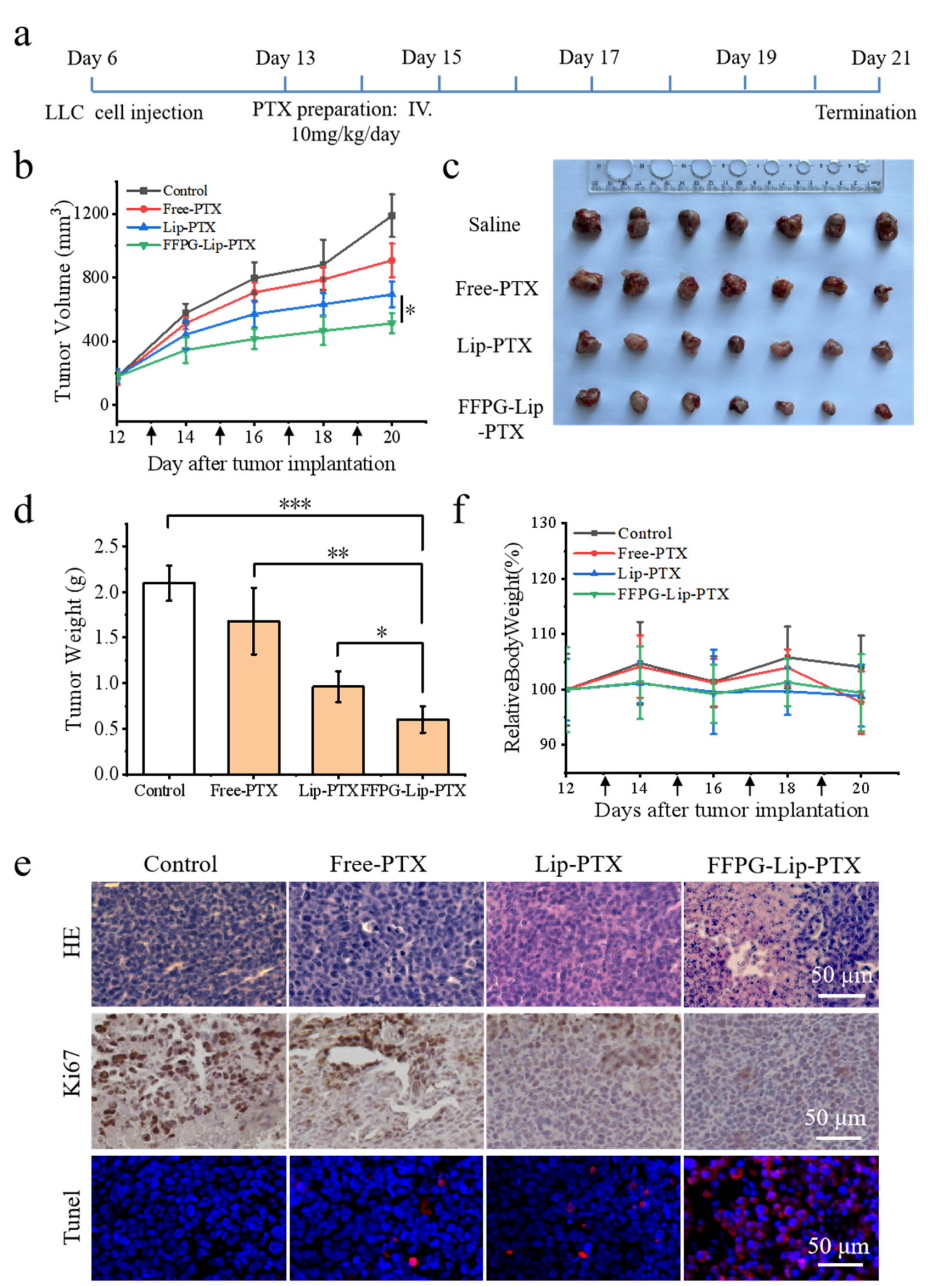
Anti-tumor effects of different PTX prescriptions (10mg/kg) on LLC tumor-bearing mouse model. (**a**) Schematic diagram of administration of mouse lung cancer-transplanted tumor model. (**b**) Tumor volume curve. (**c**) Photographs of isolated tumor tissue. (**d**) The weight of the tumor removed. (**e**). H&E pathological images of tumor tissue were resected, the proliferation images of tumor tissue were evaluated by IHC, and TUNEL fluorescence staining of apoptotic cells in tumor tissue was performed. (**f**). Weight change curve. The data are mean ± SD (n = 6), * *p* < 0.05,** *p*< 0.01. *** *p* <0. 001.

The anti-tumor effects of FFPG-Lip-PTX in vivo were further explored. The experimental design is illustrated in Figure 6a. Combining the data of each group, FFPG-Lip-PTX showed an obvious anti-tumor effect (Figure 6b–d). Because of the specific affinity of FFPG to Rab21, FFPG-Lip-PTX actively targeted lung cancer tissues. The H&E staining results showed that there were different degrees of tumor necrosis in the administration groups, and the most significant tumor necrosis was caused by FFPG-Lip-PTX (Figure 6e). Furthermore, after the FFPG-Lip-PTX treatment, the number of Ki67 positive cells stained by immunohistochemistry (IHC) was significantly lower than that in the other groups, and the number of TUNEL-positive cells were significantly higher than that in other groups (Figure 6e). The results indicated that FFPG-Lip-PTX showed the strongest inhibition of tumor proliferation and the promotion of tumor apoptosis, which resulted in its anti-tumor effect in vivo being significantly superior to other groups. This was consistent with the results for tumor volume and weight.

During the 10-day treatment cycle, the weight of the mice was monitored, and there was a negligible variation in the average body weights in different groups, indicating that our FFPG-Lip-PTX led to no severe systemic toxicity in the mice (Figure 6f). Furthermore, neither significant damage nor inflammation was detected from the H&E examination of the main organs from the corresponding mice treated with the FFPG-Lip-PTX group, indicating that FFPG had no obvious side effect on normal organs (Figure S1a). Serum biochemical markers (Figure S1b) proved the same conclusion as above, indicating the biosafety of FFPG-Lip-PTX. The aforementioned findings revealed that that FFPG-modified liposomal complex has good biocompatibility and safety.

The lung is one of the most common sites of metastasis in most cancer patients [37]. The lung transplant tumor model we constructed also enabled us to observe the metastasis of cancer cells in the lung. Figure S1a shows that FFPG-Lip-PTX had the most significant inhibitory effect on the metastasis of a lung cancer-transplanted tumor to the lung. Metastatic nodules were scattered on the surface of the lungs of the saline-treated control group, and metastatic nodules were also scattered in the lungs of mice treated with free PTX and Lip-PTX. No obvious metastatic nodules were found in the FFPG-Lip-PTX group. As with the above reasons, differences between individuals lead to large differences in tumor sizes and metastatic nodules among individuals in the same treatment group. This provided a further confirmation of metastasis, combined with the histopathological evaluation. As shown in Figure S1a, after the H&E staining, most areas in the lung section images of the saline group and the free PTX group were scattered, with large density changes, indicating tumor metastasis. The H&E staining of lung sections in the FFPG-Lip-PTX group did not show a significant tumor burden area. In conclusion, FFPG-Lip-PTX has the potential to prevent lung colonization by other cancer cells.

**Figure 7:**
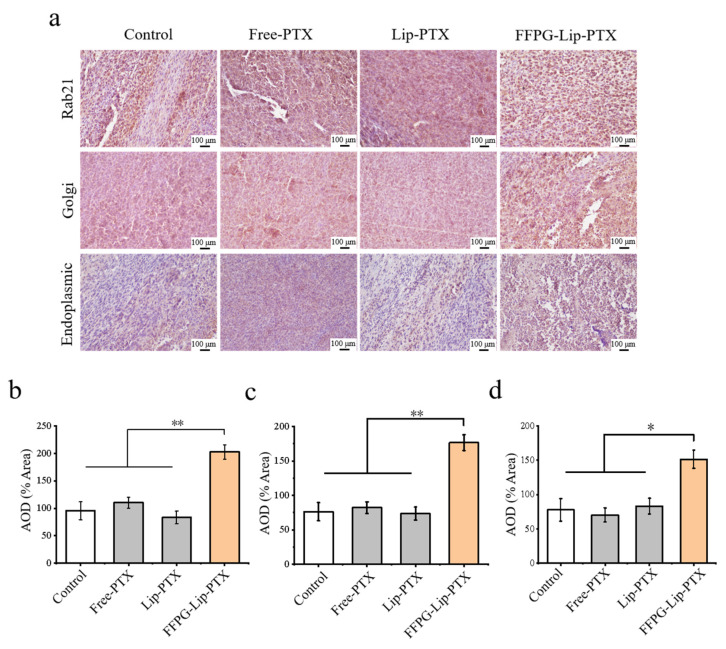
The effects of FFPG on Rab21, Golgi apparatus, and endoplasmic reticulum of lung cancer cells were observed by the immunohistochemical method. (**a**) Bright field images of Rab21, Golgi apparatus, and endoplasmic reticulum, scale 100 μm. (**b**) Rab21, (**c**) Golgi, (**d**) positive rates of endoplasmic reticulum. The data were mean ± SD (n = 6), * *p* < 0.05; ** *p* < 0.01.

In previous studies, when PTX was used alone in vivo, high doses of PTX (>40 mg/kg) were always required to produce significant anticancer activity against metastatic lung cancer [38]. However, in the present study, PTX coated with FFPG-encapsulated liposomes was effective in the intracellular treatment of lung cancer transplantation, even at a relatively low dose (10 mg/kg). This superior anti-tumor activity should be attributed to the high tumor-targeting efficacy of FFPG-Lip-PTX through passive and active targeted administration. FFPG encapsulation on the surface of liposomes enhances the specific internalization-mediated endocytosis of liposomes by FFPG/Rab21 receptors. Finally, the positive rates of Rab21, Golgi, and ER in isolated tumors in each group were detected by IHC, and the results showed that the FFPG-Lip-PTX group led to significant positive rates compared with the other three groups (Figure 7). This further confirmed our previous findings: the targeting of FFPG to lung cancer cells is achieved by the up-regulation of Rab21 and the activation of the endoplasmic reticulum–Golgi transport system. Therefore, our new findings suggest that FFPG-modified liposome nanocarriers have a specific enhancement effect on the uptake of drugs by lung cancer cells, with the potential to become a new method for lung cancer treatment. However, due to the limitations of the model, the efficacy evaluated in the manuscript cannot directly represent humans or other large animals.

## 4. Conclusions

In summary, we constructed an FFPG-Lip-PTX targeting NDDS for PTX delivery to lung cancer specifically. The physical characteristics, cellular drug function, in vivo biological distribution, anti-tumor efficacy, biosafety, and lung-targeting mechanism of FFPG-Lip-PTX were extensively characterized and tested in different models. FFPG modification improved the stability of liposomes without damaging the slower release properties. In addition, the FFPG-Lip-PTX complex was better and more quickly internalized in lung cancer cells, due to the specific affinity between FFPG and Rab21, which promoted the accumulation of FFPG-Lip-PTX into lung cancer tissues. With endocytosis, the Golgi-ER transport system was activated by FFPG, and the transport gene was up-regulated by related factors. Further accelerated FFPG-Lip-PTX was transported into the cell as a result of the overall action. Then, the cytotoxicity, anti-invasion, and anti-migration effects were enhanced. Furthermore, the biological distribution in LLC tumor-bearing mice also confirmed that FFPG-Lip-PTX led to the preferential and long-acting accumulation of tumor sites and exhibited superior anti-tumor and anti-metastatic activity to other agents.

## Data Availability

The original contributions presented in this study are included in the article/supplementary material. Further inquiries can be directed to the corresponding author.

## Supplementary Materials

The following supporting information can be downloaded at: https://www.mdpi.com/article/10.3390/pharmaceutics17010094/s1, Figure S1: The in vitro cytotoxicity of FFPG-Lip preparations at different concentrations on A549 and LLC cells; Figure S2: The biosafety of different PTX prescriptions (10 mg/kg) on LLC tumor-bearing mouse model.
