# Supplementary material for: Rab21-Targeted Nano Drug Delivery System-Based FFPG for Efficient Paclitaxel Delivery to Inhibit Lung Cancer Progression
Source: Pharmaceutics. 2025 Jan 12;17(1):94. doi: 10.3390/pharmaceutics17010094 (PMC11768108; doi:10.3390/pharmaceutics17010094)
Supplement: Supplementary file 1 [file pharmaceutics-17-00094-s001.zip › pharmaceutics-3354658-supplementary.pdf]

Supplementary Materials:

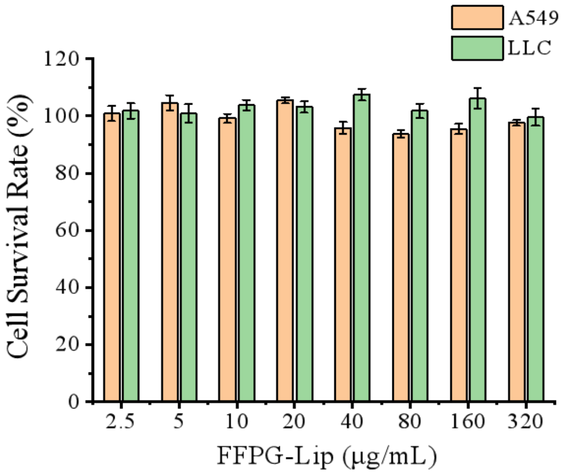

Figure S1. The in vitro cytotoxicity of FFPG-Lip preparations at different concentrations on (a) A549 and (b) LLC cells was determined by SRB method.

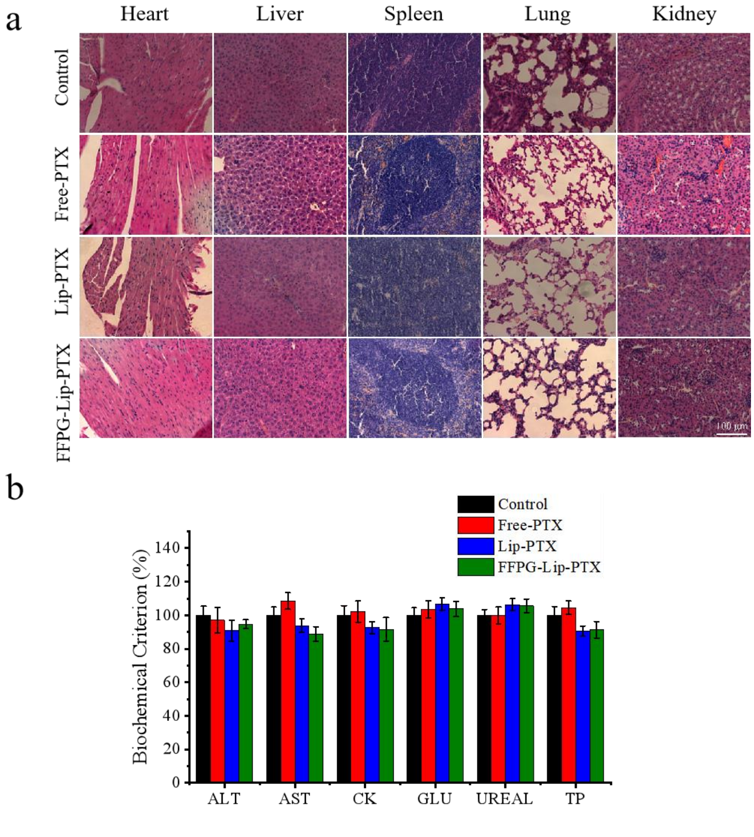

Figure S2. The biosafety of different PTX prescriptions (10mg/kg) on LLC tumor-bearing mouse model .(a). H&E staining photographs of main organs from different PTX treatment groups. (b). Serum main biochemical indexes of mice in different PTX treatment groups.
